# The Implication of Dropping Race from the MDRD Equation to Estimate GFR in an African American-Only Cohort

**DOI:** 10.1155/2021/1880499

**Published:** 2021-11-16

**Authors:** Ernie Yap, Yelyzaveta Prysyazhnyuk, Jie Ouyang, Isha Puri, Carla Boutin-Foster, Moro Salifu

**Affiliations:** Department of Medicine, SUNY Downstate Health Sciences University, Brooklyn, NY, USA

## Abstract

The widely used Modification of Diet in Renal Disease (MDRD) formula adapts a 1.212 multiplier for individuals who are identified as African Americans (AAs) or Blacks, which leads to a higher GFR estimation. As it stands, AAs have a lower prevalence of chronic kidney disease (CKD) but higher incidence of end-stage renal disease (ESRD) compared with Whites. Many hypotheses have been postulated to explain this paradox, but the imprecision of the GFR estimation with race-adaptation could be contributory. We performed a single-center, longitudinal, retrospective study on a cohort of outpatient AA patients using the MDRD and MDRD_race removed_ and CKD-EPI and CKD-EPI_race removed_ and their progression to CKD G5 (eGFR <15 ml/min/1.73 m^2^). 327 patients were analyzed. Median follow-up was 88.1 months (interquartile range, 34.4–129.1). When race was removed from MDRD, 39.9% of patients in CKD G1/2 were reclassified to CKD G3a, 72.6% of patients in CKD G3a would be reclassified to CKD G3b, and 54.1% and 36.4% of patients would be reclassified from CKD 3b to CKD G4 and CKD G4 to CKD G5, respectively (*p* < 0.0001). Comparing the CKD-EPI formula against the MDRD in our cohort, we found that 8.2%, 18.8%, and 11.4% of patients were reclassified from CKD G1/2 to CKD G3a, CKD G3a to G3b, and CKD G3b to CKD G4 respectively. Overall median time to progression to CKD G5 was 137.4 (131.9–142.8) months in patients who were not reclassified and 133.6 (127.6–139.6) months for patients who were reclassified by MDRD_race removed_(*p* < 0.288). Concerns of inequitable access to healthcare have elicited calls to review race-corrected eGFR equations. A substantial number of individuals would have their CKD stage reclassified should have the MDRD_race removed_ equation be adopted *en masse* on an AA-only population. The discrepancy is highest at the 45–59 and >60 ml/min/1.72 min^2^ ranges. This will have tremendous impact on our center's approach to pharmacological dosing, referral system, best practices, and outcome surveillance. Comprehensive review of the current “race-corrected” eGFR will require a multifaceted approach and adjunctive use of noncreatinine-based approach.

## 1. Introduction

Chronic kidney disease (CKD) is defined as abnormalities of kidney structure or function, present for more than 3 months, with adverse implications on health. Equations that determine the estimated filtration rate (eGFR) are used universally to determine the CKD stage and guide clinical decision-making, including appropriate drug dosing, dialytic therapies, or listing for kidney transplantation. African Americans (AA) have lower prevalence of CKD [1] but higher incidence of end-stage renal disease (ESRD) compared with Whites [2]. The use of eGFR may contribute to the discrepancy between the prevalence of CKD and ESRD because these equations result in a higher reported eGFR for anyone identified as AA.

While studies have observed a higher serum creatinine in AA with CKD [3], the association of serum creatinine is not fully explained by body composition. The inclusion of race in the estimation of GFR is controversial and calls for further attention. Despite a lack of scientific evidence supporting racial genetic clusters and that race is a social construct, race as a variable continues to be inserted in biological measures [4]. Race has been integrated in the interpretation of pulmonary function, determination of need for bone mass density in Black women, and optimal weight for assessing metabolic risks in Asian adults [5].

The rationale for the use of race as a coefficient in eGFR equations is that it mitigates bias and improves performance of the equation on the population from which it is derived [6, 7]. However, this assertion was based on the assumption that all AA have higher muscle mass, which was not based on actual measurement of muscle mass [8]. As an example, the inclusion of race would consider muscle mass of a patient with muscular dystrophy equal in contribution to GFR of a person with myostatin-related muscle hypertrophy, a rare condition of reduced body fat and increased skeletal muscle size, if both were AA [9].

Accurate staging of CKD is critically important in medical care and tremendous impact in raising awareness for the individual patient and implementation of key primary and secondary preventive measures such as control of hypertension, the use of renin-angiotensin-aldosterone inhibition, sodium-glucose-transporter inhibitor therapy, and proper medication dosing [10].

The use of race does not account for heterogeneity within racial groups. Therefore, the use of a race coefficient in the estimation of diagnostic biological measures is inherently flawed and devoid of a strong scientific premise and lacks precision [8]. The inclusion of “race-corrected” eGFR essentially overestimates GFR in a large part of the population and leads to missed opportunities for early intervention, referral to treatment, and as has been shown recently, access to kidney transplantation [11]. Re-examination of GFR estimating equations within predominant AA cohorts is critical to eliminating racial inequities in CKD outcomes [12, 13].

A cross-sectional study involving 2225 AA patients showed that 33.4% of them would be hypothetically reclassified to a more severe CKD stage if the race multiplier was removed from the CKD-EPI equation [14]. In an analysis of the National Health and Nutrition Examination Survey (NHANES) data, it was found that removal of the race adjustment increased CKD diagnoses among AAs (14.9% to 18.4% (95% CI, 3.2%–3.9%)) and would have enhanced access to specialist care to these individuals. However, the authors conceded that many institutions use the Modification of Diet in Renal Disease (MDRD) equation and posited that elimination of the race correction could lead to even more individuals being reclassified due to the higher race-coefficient in MDRD [15].

The objective of this study is to determine the impact of removing the race correction from the MDRD and CKD-EPI on CKD staging and analyze the progression to CKD 5 (eGFR <15 ml/min/1.73 m^2^) in people who are reclassified by the correction removal.

## 2. Methods

### 2.1. Patients and CKD Stratification

A retrospective, longitudinal review of the electronic health record (EHR) of Nephrology AA patients from a large academic medical center located in Central Brooklyn was performed. Our center serves a predominantly AA community that has been subject to widespread disparate health outcomes [16]. The race/ethnicity makeup is as follows: White—3%, Black—86%, Hispanic—8%, and Asian—2% [17]. Residents in this community have higher rates of comorbid conditions such as hypertension, diabetes, and obesity which places them at higher risk for developing CKD. The community as a whole also has a higher poverty rate, rent burden, and uninsured individuals compared to the rest of New York City [18]. Race designation was obtained from the EHR which is a mixture of self-identification and administrative designation.

For all patients, the laboratory measurement of serum creatinine was performed using the Beckman Coulter AU2700 system (Beckman Coulter, Inc., Brea, California, USA). Serum creatinine was analyzed by the kinetic alkaline picrate methodology which is traceable to the reference method based on isotope dilution-mass spectrometry (IDMS). GFR was estimated according to the MDRD abbreviated formula [6, 19].

We used the 2012 KDIGO Clinical Practice Guideline for the Evaluation and Management of CKD, which classifies estimated GFR in the following ranges:

- Stage G1/2: −60–89 ml/min/1.73 m^2^
- Stage G3a: 45 to 59 ml/min/1.73 m^2^
- Stage G3b: 30 to 44 ml/min/1.73 m^2^
- Stage G4: 15 to 29 ml/min/1.73 m^2^
- Stage G5: less than 15 ml/min/1.73 m^2^ [20]

The following equations are used:

- MDRD: eGFR (ml/min/1.73 m^2^) = 175 × (S_Cr_/88.4)^−1.154^ × age^−0.203^ × 1.212 (if the patient is AA) × 0.742 (if female) [21].
- MDRD_race removed_: eGFR = 175 × (S_Cr_/88.4)^−1.154^ × age^−0.203^ × 0.742 (if female)
- CKD-EPI: eGFR = 141 ×  min (S_Cr_/*κ*, 1)^*α*^ ×  max(S_Cr_/*κ*, 1)^−1.209^ × 0.993^Age^ × 1.018 (if female) ×  1.159 (if AA) [22].
- CKD-EPI_race removed_: eGFR = 141 × min (S_Cr_/*κ*, 1)^*α*^ × max(S_Cr_/*κ*, 1)^−1.209^ × 0.993^Age^ × 1.018 (if female)

### 2.2. Baseline Data

Past medical history of hypertension, diabetes mellitus, congestive heart failure, stroke, coronary artery disease, and dyslipidemia and etiology of CKD and laboratory data were extracted from the EHR which is populated during in-person medical visits. The body mass index (BMI) was measured by calculating the individual's weight divided by the square of the height and expressed as kg/m^2^.

### 2.3. Outcome Data

The observation period of each patient was defined as the time period from the first registered measurement of serum creatinine until CKD G5, defined as eGFR <15 ml/min/1.73 m^2^.

### 2.4. Statistical Analyses

Continuous variables are presented as mean ± SD. Comparison of continuous variables between stages of CKD were performed using one-way ANOVA or the Kruskal–Wallis test, depending on the underlying distribution. Categorical and nominal data were compared using the Χ^2^ test. The Kaplan–Meier method was used to describe the progression to CKD G5. The log-rank test was used to compare between groups [23]. All computations were performed with SPSS 27. Data collection protocol was approved by the Research and Ethics Committee of SUNY Downstate Medical Center (no. 1516654-2).

## 3. Results

### 3.1. Demographic and Clinical Characteristics

327 AA patients were analyzed. The mean age of the cohort was 61.9 ± 14.2 years. 60% of patients were female, and 57.5% had a diagnosis of diabetes mellitus. Body mass index (BMI) was evenly distributed across all groups. Over 90% of patients had a diagnosis of hypertension across all eGFR categories (Table 1).

### 3.2. Patient Outcomes

Median follow-up was 88.1 months (interquartile range, 34.4–129.1).  Table 2 shows the change in the number of patients in their respective CKD stages in which MDRD_race removed_, CKD EPI, and CKD EPI_race removed_ are applied, with the number of patients in CKD G3b almost doubling at the expense of the earlier stages in MDRD_race removed_ compared to MDRD.

When MDRD_race removed_ was applied, 39.9% of patients in CKD 1/2 were reclassified to CKD G3a, 71.8% of patients in CKD G3a would be reclassified to CKD 3b, and 54.1% and 36.4% of patients would be reclassified from CKD G3b to CKD G4 and CKD G4 to CKD G5, respectively (*p* < 0.0001) (Table 3). When the CKD-EPI formula was applied against the MDRD in our cohort, we found that 8.2%, 18.8%, and 11.4% of patients were reclassified from CKD G1/2 to CKD G3a, CKD G3a to G3b, and CKD G3b to CKD G4, respectively, and a total of 3 patients were reclassified from CKD G4 to CKD G5 (*p* < 0.0001) (Table 4).

To further evaluate the impact of removing race multiplier which our laboratory adopted the CKD-EPI formula, we compared it with CKD-EPI_race removed_ and found that CKD stage reclassification is less severe but remained significant with 22.6%, 46.5%, and 38.3% of patients reclassified from CKD G1/2, G3a, and G3b, respectively (*p* < 0.0001) (Table 5).

**Figure 1:**
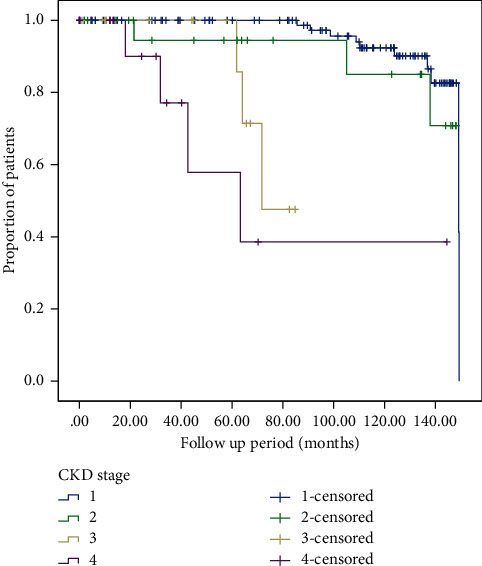
Kaplan–Meier curves depicting median time to CKD G5 amongst AA patients who were not reclassified by MDRD_race removed_ (*N* = 164).

**Figure 2:**
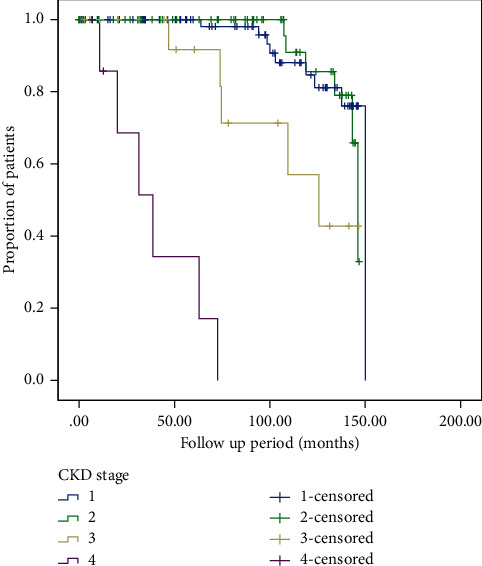
Kaplan–Meier curves depicting median time to CKD G5 amongst AA patients who were reclassified by MDRD _race removed_ (*N* = 163).

The time to progression to CKD was compared with the same patients when staged by MDRD with race compared to MDRD_race removed_. The Kaplan–Meier curves for the end point of reaching eGFR <15 ml/min/1.73 m^2^ (CKD G5) in patients with different stages of CKD who were not reclassified by MDRD_race-removed_ and those who were reclassified are depicted in Figures 1 and 2, respectively. Overall median time to progression to CKD G5 was 137.4 (131.9–142.8) months in patients who were not reclassified and 133.6 (127.6–139.6) months for patients were reclassified after removal of race (*p* < 0.288). Advanced age was a significant predictor of progression to CKD G5 (Table 6). Overall median time to CKD G5 for all patients was 135 (131.6–139.6) months (Supplementary Figure 1).

## 4. Discussion

Our analysis demonstrated the impact of removing race from MDRD and CKD-EPI equations in an AA-only patient population. In both formulas, when the race multiplier was removed, a substantial number of patients would be reclassified to higher CKD stages. Patients who were reclassified were significantly older and trended towards a more rapid progression to CKD G5.

Our results are in line with the works of Ahmed et al. [14] and Diao et al. [15] who reported similar reclassification rates from larger datasets. On an intuitive level, this finding is not surprising in which removal of a positive multiplier would result in a smaller mathematical numeral. However, in our study, we showed that patients who were reclassified by MDRD_race removed_ were significantly older in comparison with those who were not (Table 6), yet the median time to CKD G5 was not significantly shorter than their younger counterparts. This affirms the role of age in the eGFR equations and contributes to the ongoing, no-less profound debate on CKD-reclassification with respect to age; whether some people having lower renal mass as part of the normal aging process should be regarded as suffering from CKD [24]. It also implies that inclusion of race in the estimation of GFR may have greater impact on older adults. As well, in our cohort, clinical predictors such as past medical history and etiology of CKD are not a significant influence of reclassification. Tellingly, a substantial percentage (71.8%) of our patients would be reclassified from stage G3a (eGFR between 45 and 59 ml/min/1.73 m^2^) to stage G3b (eGFR between 30 and 44 ml/min/1.73 m^2^) (Table 3). In a study involving 1,120,295 adults with eGFR classified by the MDRD equation, Go et al. found that risks of death, cardiovascular events, and hospitalization were increased sharply for people with eGFR less than 45 ml/min/1.73 m^2^, denoting that the clinical importance of the accuracy is determining this cutoff [25].

Our hospital laboratory continues to report eGFR using the MDRD equation, which formed the foundation of this study. The MDRD study equation has been shown to be accurate in the low GFR range (CKD stages G3 and G4), but it underestimates GFR in higher GFR ranges (i.e., in healthy subjects) [26]. In recognition of the greater accuracy of CKD-EPI [27], due to its improvement in bias, we simulated CKD-EPI on our cohort, compared it with CKD-EPI_race removed_ (Table 5), and showed lower reclassification rates across all eGFR ranges.

In a cohort of 533 healthy Black Africans using a cutoff of 75 ml/min/1.73 m^2^, the 4-variable MDRD equation without correction for race classified the highest proportion of participants under the age of 40 years (7.8%) as having reduced eGFR [28]. In 2001, in recognition of the greater serum and urine creatinine levels amongst participants in the African American Study of Hypertension and Kidney Disease (AASK) cohort, Lewis et al. derived a five-variable eGFR formula [29]. However, the Lewis equations have never been used in clinical practice, likely due to subsequent widespread adoption of the MDRD formula. A study involving Black Africans from Ghana concluded that correction for race was unnecessary for both the MDRD and CKD-EPI equations [30]. This study compared eGFR with creatinine clearance, and the mean weight of the participants was 54.4 kg. In a cohort of 100 Black South Africans in which eGFR was compared with measured GFR (mGFR), Deventer et al. concluded that the race correction in the MDRD equation resulted in a median positive bias of 13.1 (95% CI 5.5 to 18.3) ml/min/1.73 m^2^, in comparison with a median bias of 1.9 (95% CI 0.8 to 4.5) ml/min/1.73 m^2^ when the race factor was discarded [31]. It should be noted that the mean weight of participants of the above studies was 54.5 kg and 69.6 kg, respectively. For comparison, the mean weight of participants in the AASK and MDRD cohorts was 89 kg and 79.6 kg, respectively [21, 32]. In a study that compared MDRD and CKD-EPI against mGFR in African Europeans originating from West Africa, a correction factor of 1.08 was derived [33]. Hence, the “race-correction” is in fact a reflection of assumptions of muscle mass, and consequently, the limitations surrounding the accuracy of these assumptions across swathes of population or even within an evolving population due to dietary changes will continue to hamper any formula's accuracy. Uribarri et al. recently showed that an oft-used clinical metric, the urine anion gap, can no longer be used reliably due to changes in diet across time resulting in variations to urine electrolytes [34].

In this study, the exclusion of race in both MDRD_race removed_ and CKD-EPI_race removed_ reclassified over 49.5% and 30.6% of our AA patients to a higher stage of CKD, respectively, in comparison with their conventional counterparts. This partially explains the seemingly paradoxical observation that although AA patients present with more advanced CKD stage than Whites, AAs on hemodialysis have a lower mortality than their White counterparts probably because once they are recognized within the healthcare system as dialysis patients, and eGFR becomes a less important determinant of healthcare resource [35]. Other factors such as neighborhood socioeconomic status have also been implicated [36].

Our study has the following limitations: we did not have mGFR, and as such, our comparison of various eGFR equations is not validated against a gold standard. Another major limitation is the absence of proteinuria data. As well, the definition of AA is a heterogenous one as our patient cohort comprises mainly Black individuals who derived their ancestry from the Caribbean [37]. As well, our dataset most likely suffers from lack of concordance between self-reported ethnicity and administrative race categorization due to the nature of EHRs [38, 39]. Given its retrospective nature, it is plausible that the progression to eGFR <15 ml/min/1.73 m^2^ has inherent biases. Thus, our results do not inform the biological impact of removing the race correction in eGFR formulas but highlight the disparate classification that informs clinical decision-making paradigms in our center such as referral to specialists, eligibility for kidney transplantation, and other social and disability support.

In conclusion, the removal of race multiplier factor in eGFR formulas is highly impactful on CKD epidemiology and management. The identification of an adjunctive noncreatinine-based novel biomarker remains a critical unmet medical need.

## Figures and Tables

**Table 1:** Patient characteristics.

Characteristics	Total (*N* = 327)	Chronic kidney disease glomerular stage by MDRD formula				*p* value
		G1/2 (*N* = 183)	G3a (*N* = 85)	G3b (*N* = 37)	G4 (*N* = 22)	
Age (year)	61.9 ± 14.2	58.6 ± 14.4	65.7 ± 12.5	66.2 ± 13.9	68.1 ± 11.5	0.0002
Female sex (%)	60.6	61.7	60.0	56.8	59.1	0.811
Body mass index (kg/m^2^)	29.7 ± 6.8	29.9 ± 7.0	29.4 ± 6.6	30.8 ± 6.4	27.1 ± 6.2	0.218
GFR, ml/min/1.73 m^2^	67.4 ± 27.2	86.2 ± 19.8	52.4 ± 4.4	37.0 ± 4.3	20.2 ± 3.5	<0.0001
*Medical history (%)*						
Hypertension	94.2	92.9	97.6	89.2	100	0.141
Diabetes	57.5	57.4	55.3	73.0	40.9	0.099
Congestive heart failure	19.3	19.7	21.2	16.2	13.6	0.828
Stroke	13.5	15.8	8.2	8.1	22.7	0.143
Coronary artery disease	24.5	24.6	22.4	35.1	13.6	0.274
Dyslipidemia	63.3	60.7	67.1	64.9	68.2	0.721

*Etiology of CKD (%)*						
Hypertensive nephrosclerosis	59.6	54.6	67.1	59.5	72.7	0.144
Diabetic kidney disease	37.9	38.8	32.9	45.9	36.4	0.574
Glomerulonephritis	7.6	9.8	5.9	2.7	4.5	0.362
Polycystic kidney disease	4.3	4.4	3.5	2.7	9.1	0.662
Others	24.2	26.8	22.4	21.6	13.6	0.514

**Table 2:** CKD staging of patients according to the respective eGFR formulas from a cohort of 327 AA patients.

eGFR (ml/min/1.73 m^2^)	Number of patients according to estimation formulas (%)			
	MDRD	MDRD_race removed_	CKD EPI	CKD EPI_race removed_
>60	183 (56)	110 (33.6)	168 (51.4)	130 (39.8)
45–60	85 (26)	96 (29.4)	86 (26.3)	84 (25.7
30–45	37 (11.3)	78 (23.9)	47 (14.4)	69 (21.1)
15–30	22 (6.7)	35 (10.7)	23 (7.)	37(11.3)
<15	0	8 (2.4)	3 (0.9)	7 (2.1)

**Table 3:** Comparison between the MDRD and MDRD_race removed_ formulas.

	MDRD_race removed_ (m/min/1.73 m^2^)					
MDRD eGFR (m/min/1.73 m^2^)		>60	45–59	30–44	15–29	<15
	>60	110	73 (39.9%)			
	45–59		23	61 (71.8%)	1	
	30–44			17	20 (54.1%)	
	15–29				14	8 (36.4%)

**Table 4:** Comparison between the MDRD and CKD-EPI formulas.

	CKD-EPI (ml/min/1.73 m^2^)					
MDRD eGFR (m/min/1.73 m^2^)		>60	45–59	30–44	15–29	<15
	>60	168	15 (8.2%)			
	45–59		69	16 (18.8%)		
	30–44		2	31	4 (10.8%)	
	15–29				19	3 (13.6%)

**Table 5:** Comparison between the CKD-EPI and CKD-EPI_race-removed_ formulas.

	CKD-EPI _race-removed_ (ml/min/1.73 m^2^)					
CKD-EPI eGFR (ml/min/1.73 m^2^)		>60	45–59	30–44	15–29	<15
	>60	130	38 (22.6%)			
	45–59		46	40 (46.5%)		
	30–44			29	18 (38.3%)	
	15–29				19	4 (17.4%)
	<15					3

**Table 6:** Comparison of AA patient characteristics of those were reclassified after removal of the race multiplier.

Characteristics	Chronic kidney disease glomerular stage by MDRD formula		*p* value
	Patients who were reclassified	Patients who were not reclassified	
Age (years)	64.3 ± 12.7	59.6 ± 15.2	0.003
Female sex (N)	103	95	0.330
Body mass index (kg/m^2^)	29.4 ± 6.4	29.9 ± 7.2	0.514
GFR (ml/min/1.73 m^2)^	52.5 ± 14.3	75.1 ± 28.0	<0.0001
*Past medical history (N)*			
Hypertension	157	151	0.101
Diabetes	94	94	0.949
Congestive heart failure	34	29	0.467
Stroke	21	23	0.762
Coronary artery disease	46	34	0.115
Dyslipidemia	104	103	0.851

*Etiology of chronic kidney disease (N)*			
Hypertensive nephrosclerosis	101	94	0.392
Diabetic kidney disease	67	57	0.237
Glomerulonephritis	10	15	0.306
Polycystic kidney disease	4	10	0.104
Others	34	45	0.165

## Data Availability

The data used to support the findings of this study are available from the corresponding author upon request.

## Supplementary Materials

Supplementary Materials
*Supplementary Figure 1.* Kaplan–Meier curves depicting mean duration to progression to CKD 5 in all patients by CKD stage according to MDRD.
