# Supplementary material for: The Implication of Dropping Race from the MDRD Equation to Estimate GFR in an African American-Only Cohort
Source: Int J Nephrol. 2021 Nov 16;2021:1880499. doi: 10.1155/2021/1880499 (PMC8610663; doi:10.1155/2021/1880499)

**Supp Figure 1. Kaplan Meier curves depicting mean duration to progression to CKD 5 in all patients by CKD stage according to MDRD**


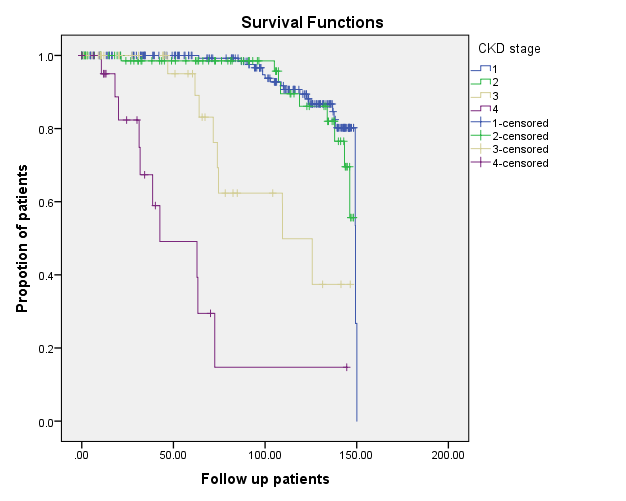

Supplement: Supplementary Materials — Supplementary Figure 1. Kaplan–Meier curves depicting mean duration to progression to CKD 5 in all patients by CKD stage according to MDRD. [file 1880499.f1.docx]
